# PEGylation of polypropylenimine dendrimers: effects on cytotoxicity, DNA condensation, gene delivery and expression in cancer cells

**DOI:** 10.1038/s41598-018-27400-6

**Published:** 2018-06-20

**Authors:** Sukrut Somani, Partha Laskar, Najla Altwaijry, Paphitchaya Kewcharoenvong, Craig Irving, Gillian Robb, Benjamin S. Pickard, Christine Dufès

**Affiliations:** 1 Strathclyde Institute of Pharmacy and Biomedical Sciences, University of Strathclyde, 161 Cathedral Street, Glasgow, G4 0RE United Kingdom; 2 Department of Pure and Applied Chemistry, University of Strathclyde, 295 Cathedral Street, Glasgow, G1 1XL United Kingdom

## Abstract

Diaminobutyric polypropylenimine (DAB) dendrimers have been shown to be highly efficient non-viral gene delivery systems for cancer therapy. However, their cytotoxicity currently limits their applications. To overcome this issue, PEGylation of DAB dendrimer, using various PEG molecular weights and dendrimer generations, has been attempted to decrease the cytotoxicity and enhance the DNA condensation, size and zeta potential, cellular uptake and transfection efficacy of these dendriplexes. Among all the PEGylated dendrimers synthesized, generation 3- and generation 4-DAB conjugated to low molecular weight PEG (2 kDa) at a dendrimer: DNA ratio of 20:1 and 10:1 resulted in an increase in gene expression on almost all tested cancer cells lines (by up to 3.2-fold compared to unmodified dendrimer in A431 cells). The highest level of β-galactosidase gene expression (10.07 × 10^−3^ ± 0.09 × 10^−3^ U/mL) was obtained following treatment of B16F10-Luc cells with G4-dendrimer PEGylated with PEG2K at a dendrimer: DNA ratio of 20:1. These delivery systems significantly decreased cytotoxicity on B16F10-Luc cells, by more than 3.4-fold compared to unmodified dendrimer. PEGylated generations 3- and 4-DAB dendrimers are therefore promising gene delivery systems for cancer therapy, combining low cytotoxicity and high transfection efficacy.

## Introduction

Gene therapy has become one of the most intensively studied strategies for the treatment of various diseases, ranging from monogenic diseases such as cystic fibrosis to complex disorders such as cancer^1^. Despite numerous advances, the use of therapeutic genes in cancer treatment is still limited by the lack of safe and efficacious gene delivery vectors^2^. To overcome this problem, various non-viral vectors, such as cationic liposomes and cationic polymers, are currently under development, due to advantages such as their simplicity to use, ease of production and quality control, high DNA carrying capacity, low immunogenicity and their ability to achieve prolonged exogenous gene expression^3^. Among these non-viral delivery systems, dendrimers appear to be particularly promising, owing to their well-defined size and structure, low polydispersity and high transfection efficiency^4–6^. In particular, diaminobutyric polypropylenimine (DAB) dendrimer has been demonstrated to be an efficient non-viral vector for targeted gene delivery to cancer^7–11^ and to the brain^12,13^. However, DAB dendrimer shows concentration- and generation-dependent toxicity, caused by the presence of surface primary amines^14^, which impedes their clinical development. In addition, dendrimers and other cationic polymers are rapidly eliminated from the systemic circulation by the mononuclear phagocyte system, thus reducing their efficacy^14^.

PEGylation, the conjugation of polyethylene glycol (PEG) to cationic polymers, is one of the most widely used strategies to shield positive charges and therefore reducing toxicity, macrophage uptake, aggregation and opsonisation, ultimately improving circulation times^15^. However, positively charged surface primary amines of the dendrimer are essential to electrostatically bind to the negatively charged phosphate groups of DNA^4^. PEGylation of these surface primary amines could modify the physicochemical properties of the dendrimer, thus impacting on its interactions with DNA and its transfection efficacy. Previous studies using generation 5-polyamidoamine (PAMAM) dendrimer demonstrated that the modification of this dendrimer with PEG 3.4 kDa resulted in a 20-fold increase in transfection efficacy compared to naked PAMAM dendrimer in Chinese hamster ovarian (CHO) cells^16^. However, modification of the same dendrimer with PEG having a different molecular weight (550 Da) led to a decrease in transfection efficiency in the same cell line^17^. Both studies reported a decrease in the cytotoxicity of the dendrimer, but an opposite outcome regarding gene expression. PEGylation of dendrimers and its impact on transfection efficacy appear to be governed by various factors such as dendrimer generation, PEG molecular weight, degree of PEGylation and tested cell lines.

The aim of this study is therefore to investigate the influence of the conjugation of PEG with various molecular weights to DAB dendrimers with various generations, on the cytotoxicity, physicochemical properties, DNA condensation, cellular uptake and transfection efficacy of the dendriplexes.

## Results

### Synthesis of PEGylated PPI dendrimers

^1^H NMR confirmed the synthesis of PEGylated DAB dendrimers. For representation we have shown the synthesis of G3-DAB dendrimer conjugated to M-PEG2K (Supplementary Fig. 1). The characteristic peaks of G3-DAB were ^1^H-NMR (D_2_O): δ DAB (N-CH_2_-**CH**_**2**_**-CH**_**2**_-CH_2_-N) = 1.57; δ DAB (N-CH_2_-**CH**_**2**_-CH_2_-N) = 1.64; δ DAB (N-CH_2_-**CH**_**2**_-CH_2_-NH_2_) = 1.79; δ DAB (N-CH_2_-CH_2_-**CH**_**2**_-NH) = 2.48; δ DAB (N-**CH**_**2**_-CH_2_-**CH**_**2**_**-**N) = 2.55; δ DAB (N-CH_2_-CH_2_-**CH**_**2**_**-**NH_2_) = 2.91. The characteristic peaks of the protons of the succinimidyl groups of M-PEG2K could be seen at 2.74. Other peaks of M-PEG2K are ^1^H-NMR (D_2_O): δ M-PEG2K (**CH**_**3**_-O-) = 3.34; δ M-PEG2K (**CH**_**2**_**-CH**_**2**_-O) = 3.66; δ M-PEG2K (**CH**_**2**_-C=O) = 4.1. The final product G3-PEG2K presented the characteristic peaks from both G3-DAB and M-PEG2K. The protons of the succinimidyl group of M-PEG2K disappeared in the final product due to the formation of amide bond with the primary amine groups of G3 DAB dendrimer. The characteristic peak of the methylene protons next to the amide bond was observed at 3.89 ppm.

The number of PEG chains attached to each dendrimer was calculated by ratios of integration between peaks at ~1.57 and 3.33 (Supplementary Figs 2–4). The number of M-PEG2K chains attached to G3-, G4- and G5-DAB dendrimers was 2.7, 2.7 and 2 respectively, whereas the number of M-PEG5K chains attached to the G3, G4 and G5 DAB dendrimers was 5.3, 2.4, 3.7 respectively, and the number of M-PEG10K chains attached to G3, G4 and G5 DAB dendrimers was 6.2, 2.3 and 6.2 respectively.

### Impact of PEGylation on cytotoxicity

**Figure 1:**
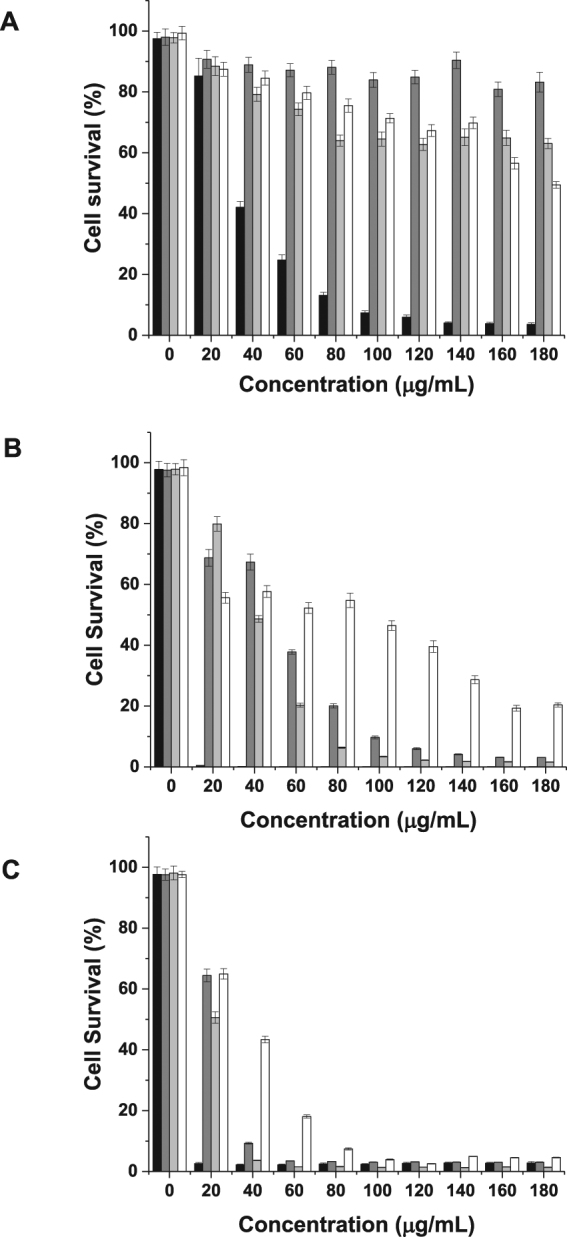
Cytotoxicity of (**A**) generation 3-, (**B**) generation 4- and (**C**) generation 5- DAB dendrimers conjugated with PEG of various molecular weights, on B16F10-Luc cancer cells (n = 15) (black: dendrimer without PEG, dark grey: dendrimer conjugated with PEG 2 kDa, light grey: dendrimer conjugated with PEG 5 kDa, white: dendrimer conjugated with PEG 10 kDa).

Dendrimers with free peripheral primary amine groups are widely known to exhibit concentration- and generation-dependent cytotoxicity. The conjugation of PEG with various molecular weights led to a decrease in the cytotoxicity of all DAB dendrimers on B16F10-Luc cells (Table 1 and Fig. 1). The conjugation of PEG with various molecular weights to G3-DAB dendrimer resulted in a minimum of 5.3-fold decrease in cytotoxicity (IC_50_ for unmodified G3-dendrimer: 33.84 ± 2.13 μg/mL, IC_50_ for G3-DAB conjugated to M-PEG2K, M-PEG5K, M-PEG10K higher than 180 μg/mL). These PEGylated dendrimers were the least cytotoxic among all those tested, with more than 50% cells being still viable at the highest tested concentration (180 μg/mL) (Fig. 1A).

**Table 1:** Cytotoxicity of generation 3-, generation 4- and generation 5- DAB dendrimers conjugated with PEG of various molecular weights, expressed as IC_50_ values, on B16F10-Luc cancer cells (n = 15).

Dendrimer generation	IC_50_ (µg/mL) (mean ± S.E.M.)			
	No PEG	PEG-2K	PEG-5K	PEG-10K
G3	33.84 ± 2.13	>180	>180	>180
G4	16.34 ± 0.51	55.49 ± 1.70	40.96 ± 1.18	>60
G5	6.04 ± 0.21	22.97 ± 0.67	21.65 ± 0.41	55.05 ± 2.38

The conjugation of PEG to G4-DAB, which was more cytotoxic than G3-DAB, led to a decrease in cytotoxicity by more than 2.5-fold (IC_50_ for unmodified G4-dendrimer: 16.34 ± 0.51 μg/mL, IC_50_ for G4-DAB conjugated to M-PEG2K, M-PEG5K, M-PEG10K: respectively 55.49 ± 1.70 μg/mL, 40.96 ± 1.18 μg/mL and higher than 60 μg/mL). PEGylation decreased cytotoxicity, but with limited efficacy, as only a maximum of 20% of cells were still viable at a treatment concentration of 180 μg/mL (Fig. 1B).

The conjugation of PEG to G5-DAB, which was the most cytotoxic of the 3 unconjugated dendrimers, led to a limited decrease of cytotoxicity, with M-PEG10K being the most efficacious (IC_50_ for unmodified G5-dendrimer: 6.04 ± 0.21 μg/mL, IC_50_ for G5-DAB conjugated to M-PEG10K: 55.05 ± 2.38 μg/mL). Less than 5% of the cells were still viable at a treatment concentration of 180 μg/mL, independently of the molecular weight of PEG used for conjugation (Fig. 1C).

### Impact of PEGylation on DNA condensation efficiency

**Figure 2:**
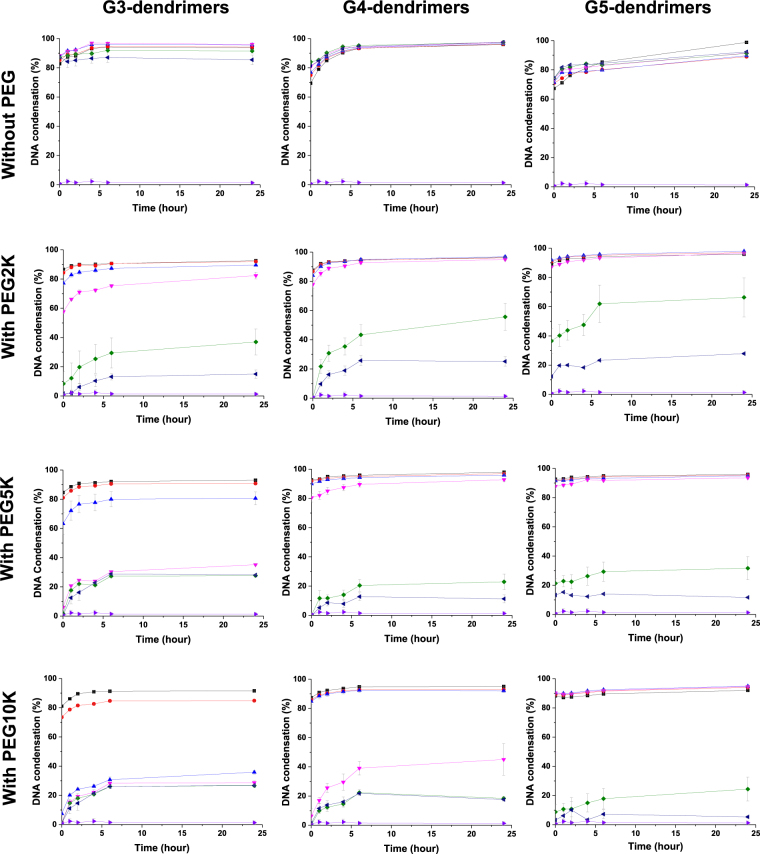
DNA condensation of generation 3-DAB (left), generation 4-DAB (middle) and generation 5-DAB (right), unmodified or conjugated with PEG2K, PEG5K or PEG10K, at various durations and dendrimer: DNA weight ratios: 20:1 (■, black), 10:1 (●, red), 5:1 (▲, blue), 2:1 (▼, pink), 1:1 (♦, green), 0.5:1 (◂, navy) and DNA (▶, purple). Results are expressed as mean ± SEM (n = 4).

All unmodified DAB dendrimers were able to efficiently condense at least 70% of negatively charged plasmid DNA to form dendriplexes, at all dendrimer: DNA weight ratios (Fig. 2). DNA complexation occurred instantly and was stable for 24 hours.

The conjugation of M-PEG2K, M-PEG5K, M-PEG10K to G3-DAB dendrimer led to a decrease in the DNA condensation efficiency. DNA condensation efficiency of G3- PEGylated dendrimers decreased when the molecular weight of conjugated PEG increased: G3-PEG2K was able to condense more than 70% of the DNA at the weight ratios of 5:1 and higher. However, a similar DNA condensation percentage could only be reached with G3-PEG5K and G3-PEG10K at the dendrimer: DNA weight ratios of 10:1 and 20:1.

PEGylation of G4-DAB dendrimer followed a similar pattern, but with a generally enhanced DNA condensation compared to what observed with PEGylated G3-DAB dendrimers. G4-PEG2K and G4-PEG5K were able to condense more than 70% of the DNA at the weight ratios of 2:1 and higher (compared to the 5:1 and 10:1 ratios needed in the case of G3-dendrimer). G4-PEG10K was less effective, with a similar DNA condensation percentage only reached at the dendrimer: DNA weight ratios of 5:1 and above.

PEGylation of G5-DAB dendrimer resulted in an even further enhanced DNA condensation compared to that observed with PEGylated G4-DAB dendrimers. G5-PEG2K and G5-PEG5K were as effective as G4-PEG2K and G4-PEG5K, but G5-PEG10K was able to condense more than 70% of the DNA at the dendrimer: DNA weight ratios of 2:1 and higher.

### Impact of PEGylation on size and zeta potential of dendriplexes and dendrimers

**Figure 3:**
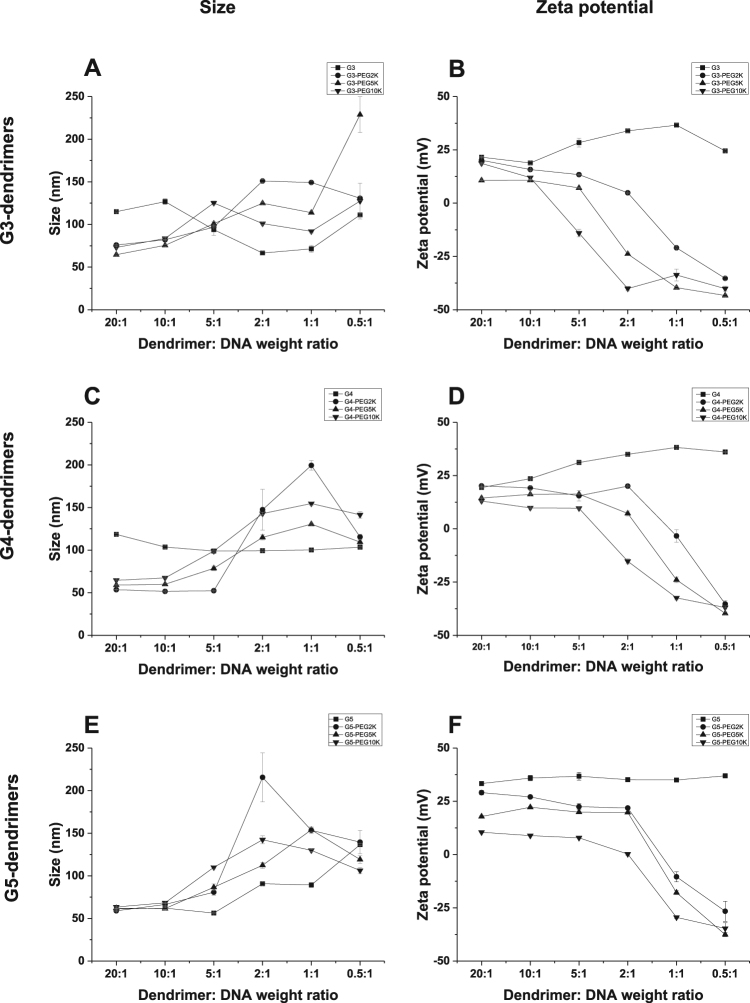
Size (left) and zeta potential (right) of the various generations of DAB dendriplexes and their PEGylated counterparts (top: generation 3-, middle: generation 4- and bottom: generation 5-dendriplexes) at various dendrimer: DNA weight ratios (20:1, 10:1, 5:1, 2:1, 1:1, 0.5:1). Results are expressed as mean ± SEM (n = 4).

The conjugation of M-PEG2K, M-PEG5K and M-PEG10K to G3- and G4-DAB dendrimers led to a decrease in the size of the dendriplex at the high dendrimer: DNA weight ratios of 20:1 and 10:1 (Fig. 3). The same conjugation on G5-DAB dendrimer did not result in any significantly different size change of the dendriplex. At low dendrimer: DNA weight ratios (from 2:1 and below), all PEGylated dendriplexes showed an increased size due to the weaker interactions between PEGylated DAB dendrimers and anionic plasmid DNA.

The conjugation of M-PEG2K, M-PEG5K and M-PEG10K to DAB dendrimers resulted in a decrease in the zeta potential of all dendriplexes at all the tested dendrimer: DNA weight ratios (Fig. 3). The decrease in the zeta potential was directly proportional to an increase in the molecular weight of PEG: zeta potential, was the lowest following conjugation of PEG10K for all 3 G3-, G4- and G5-DAB dendrimers. At low dendrimer: DNA weight ratios (2:1 and below), negative zeta potentials were recorded, due to insufficient binding between the PEGylated dendrimers and pDNA.

The size of all PEGylated dendrimers was slightly higher than that of non-PEGylated dendrimers (Supplementary Table 1). For G3-DAB dendrimer, it increased with the size of the conjugated PEG chains (1.73 ± 0.02 nm for non-PEGylated G3-DAB, from 3.14 ± 0.16 nm for G3-PEG2K to 6.1 ± 0.13 nm for G3-PEG10K). For G4-DAB dendrimer, the size of the dendrimer mainly increased following conjugation of M-PEG5K and M-PEG10K (2.01 ± 0.04 nm for non-PEGylated G4-DAB, 5.25 ± 0.72 nm and 6.54 ± 0.27 nm for G4-PEG5K and G4-PEG10K respectively). For G5-DAB dendrimer, it was the highest following grafting with M-PEG10K (2.53 ± 0.07 nm for G5-DAB, 6.43 ± 0.24 nm for G5-PEG10K).

The zeta potential of the PEGylated dendrimers was significantly lower than that of non-PEGylated dendrimers (with the exception of G3-dendrimer and G3-DAB2K). Grafting M-PEG10K to G3-, G4- and G5-dendrimers led to the lowest zeta potential among PEGylated dendrimers (8.41 ± 0.42 mV, 8.74 ± 0.14 mV and 10.3 ± 0.22 mV respectively for G3–10K, G4–10K and G5–10K, 13.05 ± 0.59 mV, 25.26 ± 0.78 mV and 41.5 ± 1.52 mV respectively for G3-, G4- and G5-DAB) (Supplementary Table 2).

### Impact of PEGylation on transfection

**Figure 4:**
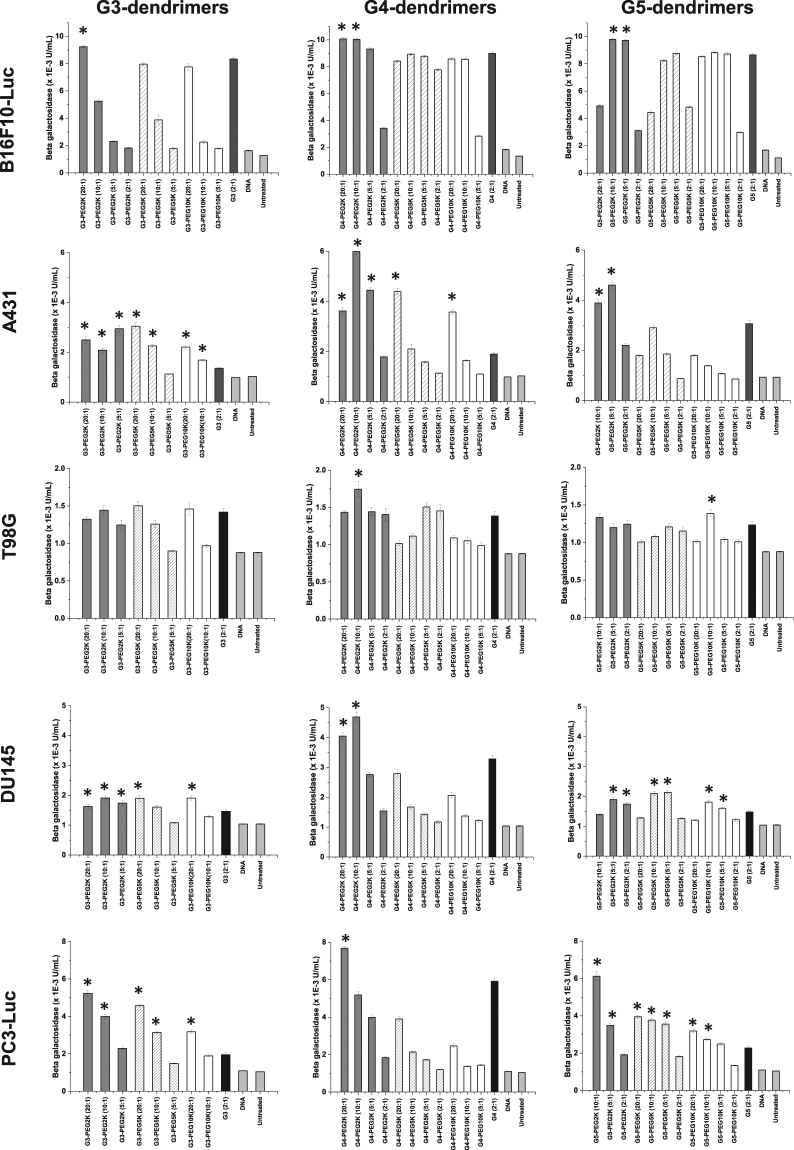
Transfection efficacy of various generations of DAB dendriplexes and their PEGylated counterparts at various dendrimer: DNA weight ratios in B16F10-Luc, A431, T98G, DU145 and PC3-Luc cancer cell lines. Results are expressed as the mean ± SEM of three replicates (n = 15). *P < 0.05 vs the transfection levels of unmodified G3-, G4- and G5-dendriplexes. Significance is only indicated on the figure if the transfection level of PEGylated dendriplex is higher than their unmodified counterpart.

The PEGylation of DAB dendrimers led to an increase in transfection for some specific generation-dependent dendrimers, PEG molecular weight, dendrimer: DNA weight ratio, and depending on the cell line (Fig. 4). This transfection increase did not follow any trend applicable to all dendrimers with one given PEG.

PEGylated G3-dendrimers led to a transfection increase (by 1.1-fold compared to unmodified dendrimer) only following conjugation of PEG2K, at a dendrimer: DNA weight ratio of 20:1 on B16F10-Luc cells. The highest gene expression obtained in PC3-Luc cells (increase of 2.7-fold compared to unmodified dendrimer) was also obtained with the same conditions. However, on A431 cells, gene expression was increased for almost all tested PEG molecular weights and dendrimer: DNA weight ratios (up to 2.2-fold), except when using M-PEG5K at a dendrimer: DNA ratio of 5:1. On T98G cells, PEGylation did not result in any transfection increase compared to the native G3-dendrimer.

G4-dendrimer PEGylated with PEG2K led to a transfection increase for all the tested cell lines at a dendrimer: DNA ratio of 20:1 and/or 10:1 (up to 1.1-fold in B16F10-Luc cells, up to 3.2-fold in A431 cells, up to 1.3-fold in T98G cells, up to 1.4-fold in DU145 cells, up to 1.3-fold in PC3-Luc cells compared to unmodified dendrimer). On A431 cells, conjugation of PEG5K and 10 K could lead to a more modest transfection increase (by 2.3-fold and 1.9-fold respectively), when used at a dendrimer: DNA ratio of 20:1.

G5-dendrimer conjugated to PEG2K resulted in a transfection increase as well on B16F10-Luc (up to 1.1-fold), A431 (up to 1.5-fold), DU145 (up to 1.4-fold) and PC3-Luc cells (up to 2.7-fold), but not on T98G cells. This increase was observed at various dendrimer: DNA weight ratios, depending on the cell line. On T98G, DU145 and PC3-Luc cells, the conjugation of PEG with a molecular weight of 5 or 10 K resulted as well in an increase in transfection, for some specific dendrimer: DNA ratios (up to 1.1-fold in T98G cells, up to 1.4-fold in DU145 cells, up to 1.7-fold in PC3-Luc cells).

The highest transfection increase was therefore obtained following treatment of A431 cells with G4-dendrimer PEGylated with PEG2K at a dendrimer: DNA ratio of 10:1 (up to 3.2-fold compared to unmodified dendrimer). The highest level of β-galactosidase gene expression (10.07 × 10^−3^ ± 0.09 × 10^−3^ U/mL) resulted from the treatment of B16F10-Luc cells with G4-dendrimer PEGylated with PEG2K at a dendrimer: DNA ratio of 20:1.

### Impact of PEGylation on cellular uptake

**Figure 5:**
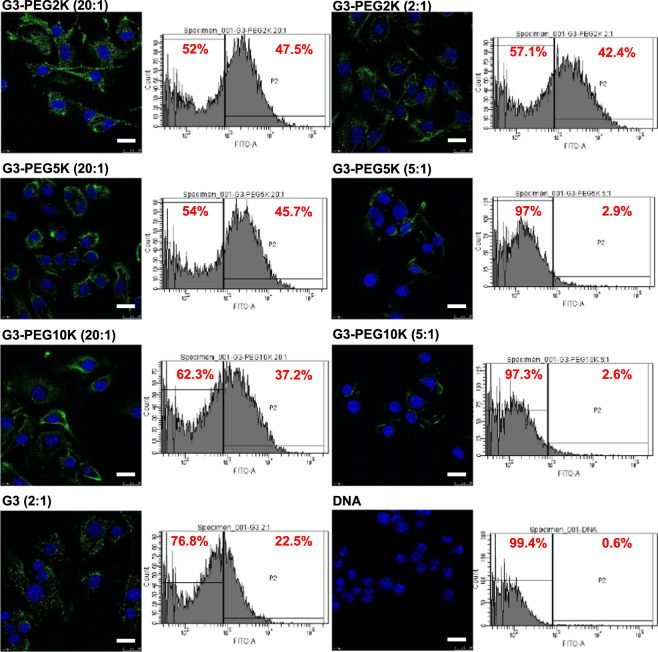
Confocal microscopy imaging and flow cytometry quantification of the cellular uptake of fluorescein-labelled DNA (2.5 μg/well) complexed with G3-PEG2K (dendrimer: DNA weight ratios: 10:1 and 2:1), G3-PEG5K (dendrimer: DNA weight ratios: 20:1 and 2:1), G3-PEG10K (dendrimer: DNA weight ratios: 20:1 and 5:1), G3-DAB (dendrimer: DNA weight ratio: 2:1) or left uncomplexed, after incubation for 1 h with B16F10-Luc cells (Blue: nuclei stained with DAPI (excitation: 405 nm laser line, bandwidth: 415–491 nm), green: fluorescein-labelled DNA (excitation: 543 nm laser line. bandwidth: 550–620 nm) (Bar: 10 µm).

**Figure 6:**
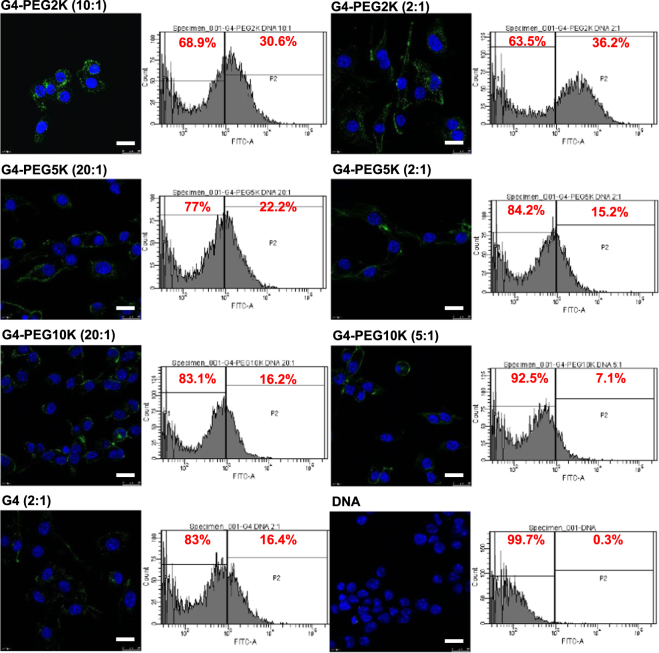
Confocal microscopy imaging and flow cytometry quantification of the cellular uptake of fluorescein-labelled DNA (2.5 μg/well) complexed with G4-PEG2K (dendrimer: DNA weight ratios: 10:1 and 2:1), G4-PEG5K (dendrimer: DNA weight ratios: 20:1 and 5:1), G4-PEG10K (dendrimer: DNA weight ratios: 20:1 and 3:1), G4-DAB (dendrimer: DNA weight ratio: 2:1) or left uncomplexed, after incubation for 1 h with B16F10-Luc cells (Blue: nuclei stained with DAPI (excitation: 405 nm laser line, bandwidth: 415–491 nm), green: fluorescein-labelled DNA (excitation: 543 nm laser line. bandwidth: 550–620 nm) (Bar: 10 µm).

**Figure 7:**
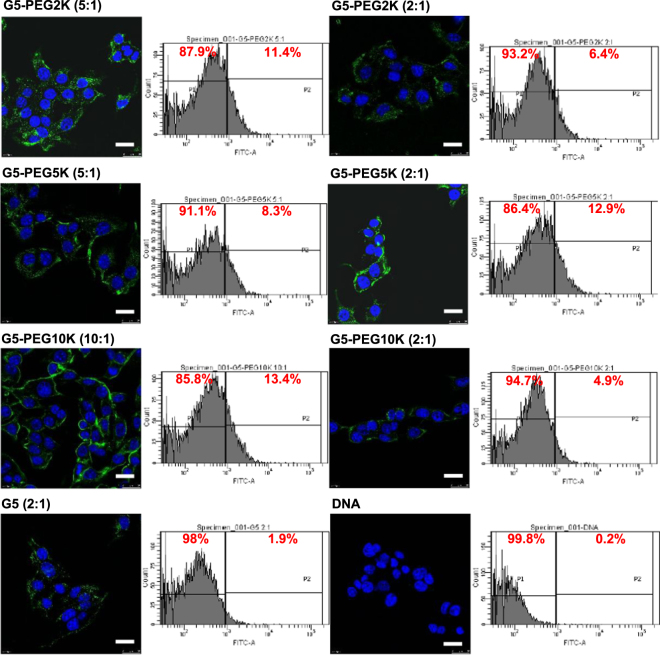
Confocal microscopy imaging and flow cytometry quantification of the cellular uptake of fluorescein-labelled DNA (2.5 μg/well) complexed with G5-PEG2K (dendrimer: DNA weight ratios: 5:1 and 2:1), G5-PEG5K (dendrimer: DNA weight ratios: 5:1 and 2:1), G5-PEG10K (dendrimer: DNA weight ratios: 10:1 and 2:1), G5-DAB (dendrimer: DNA weight ratio: 2:1) or left uncomplexed, after incubation for 1 h with B16F10-Luc cells (Blue: nuclei stained with DAPI (excitation: 405 nm laser line, bandwidth: 415–491 nm), green: fluorescein-labelled DNA (excitation: 543 nm laser line. bandwidth: 550–620 nm) (Bar: 10 µm).

To determine if there was a correlation between levels of transfection and cellular uptake of the dendriplexes, we treated B16F10-Luc cells with various dendriplexes that showed high levels and low levels of transfection and determined the cellular uptake qualitatively using confocal microscopy and quantitatively using flow cytometry. G3-PEG2K (20:1), G3-PEG5K (20:1) and G3-PEG10K (20:1) dendriplexes, that led to high levels of transfection, resulted in higher cellular uptake than that of G3-PEG2K (2:1), G3-PEG5K (5:1) and G3-PEG10K (5:1) dendriplexes respectively (Fig. 5). G4-PEG2K (10:1) dendriplex, which led to high transfection, unexpectedly showed lower cellular uptake compared to G4-PEG2K (2:1) dendriplex which led to lower transfection (Fig. 6). G5-PEG2K (5:1), G5-PEG5K (5:1) and G5-PEG10K (10:1) dendriplexes, that led to higher transfection levels, also showed moderately higher cellular uptake compared to G5-PEG2K (2:1), G5-PEG5K (2:1) and G5-PEG10K (2:1) dendriplexes that resulted in low transfection efficiency (Fig. 7).

## Discussion

To our knowledge, this is the first report methodically studying the effect of the conjugation of PEG with various molecular weights to generations 3-, 4- and 5-diaminobutyric polypropylenimine dendrimers, on their cytotoxicity, DNA condensation, size and zeta potential, transfection and cellular uptake efficacy. We have successfully conjugated M-PEG2K, M-PEG5K and M-PEG10K to the primary amine groups on the surface of G3-, G4- and G5-DAB dendrimers through an amide bond formation. These primary amines have been described to exert dose-dependent and generation-dependent toxicity through formation of nanoholes in the cellular membrane^18^.

Toxicity is one of the major limitations for using dendrimers as gene delivery systems, in particular for high generation dendrimers that theoretically lead to higher transfection. The conjugation of PEG with various molecular weights to DAB dendrimers of various generations led to a decrease in the cytotoxicity of the dendrimers by shielding their surface positive charges. Specifically, the conjugation of M-PEG2K, M-PEG5K and M-PEG10K to G3-DAB dendrimer led to the most significant decrease in cytotoxicity among all the tested dendrimers. For G4-DAB and G5-DAB dendrimers, the highest decrease in cytotoxicity was observed following conjugation of M-PEG10K. Expectedly, the size of all PEGylated dendrimers was slightly higher than that of unmodified dendrimers, but did not correlate with a specific trend regarding the decrease in cytotoxicity observed in the study. These very small sizes need to be considered with caution, as they are in limit of detection of the Zetasizer. The zeta potential of the PEGylated dendrimers was statistically lower than their non-PEGylated counterparts (with the exception of G3-dendrimer and G3-DAB2K) due to the charge-shielding effect of PEG, and therefore did have a positive impact on cytotoxicity. Cell viability has previously been reported to be negatively influenced by possible intermolecular agglomeration of the PEGylated dendrimers through twinning of long PEG chains, that occurs at high concentrations^19,20^. However, this eventual agglomeration did not impact on cytotoxicity in this study: the conjugation of high molecular weight M-PEG10K to the G5-DAB dendrimer decreased its cytotoxicity compared to the other PEGylated G5-dendrimers. This result is in accordance with similar reduction of toxicity reported when conjugating G5-PAMAM dendrimer to PEG 3.4 kDa^16^, hyperbranched PAMAM to PEG 2 kDa^21^ and G5- and G6- PAMAM to PEG 5 K^22^.

The charge-shielding effect of PEG had a substantial effect on the DNA condensation efficiency of the different generations of DAB dendrimers. Increase in the molecular weight of PEG conjugated to DAB dendrimers led to a decrease in the DNA condensation efficiency at lower dendrimer: DNA weight ratios. At higher ratios, stable dendriplex formation with DNA condensation with more than 70% DNA condensation efficiency was recorded. Hashemi *et al*.^23^ reported similar results with a slight decrease in the DNA condensation ability of the G5-DAB dendrimer modified with PEG (MW: 3.4 kDa). Furthermore, G5-PAMAM dendrimer modified with PEG (MW: 3.4 and 5 kDa) showed an increased ability to compact DNA compared to unmodified dendrimers^16,24^. These results can be explained by the fact that the conjugation of PEG to cationic dendrimers led to steric stabilization of the electrostatic interactions between the dendrimer and the plasmid DNA.

PEGylated G3- and G4-DAB dendriplexes showed a lower size compared to the non-PEGylated dendriplexes only at high dendrimer: DNA weight ratios (20:1 and 10:1). The conjugation of PEG chains imparted flexibility to the dendrimers, leading to the formation of more compact dendriplexes at high dendrimer: DNA weight ratios^25^. A similar decrease in the size of the dendriplexes was reported when G5- and G6-PAMAM dendrimers were conjugated to PEG (MW: 5 kDa)^24^. When these dendrimers were modified with PEG (MW: 3.4 kDa), the size of the resulting PEGylated dendriplexes were smaller than that of the unmodified PAMAM dendrimers^23^.

The cationic surface charge is one of the important determinants of the dendrimer-mediated cytotoxicity. PEGylation of dendrimers led to charge shielding effects, thus increasing the biocompatibility. The conjugation of PEG led to a decrease in the zeta potential of the dendriplexes compared to the unmodified dendriplexes. Similar results were reported following conjugation of PEG to various polymers^24^. A decrease in zeta potential should reduce the interactions with serum proteins and uptake by the reticuloendothelial system *in vivo*, leading to longer circulation time of the dendriplexes^26^. It should also increase the hemocompatibility of the dendriplexes^26^.

Dendriplex-mediated transfection was hindered by various rate-limiting steps such as cellular uptake, endosomal escape and nuclear entry. These rate-limiting steps are controlled by a complex interplay between the DNA condensation efficiency, size, zeta potential and surface characteristics of the dendriplexes, which ultimately depend on the generation of dendrimer and the PEG molecular weight. In this study, we reported significantly higher transfection levels by PEGylated dendrimers at higher dendrimer: DNA weight ratios (20:1 and 10:1) compared to unmodified dendrimers. The conjugation of PEG to the DAB dendrimers imparts higher flexibility and steric stabilization of the complexes. In addition, PEGylation creates charge separation between the positively charged dendrimers and negatively charged DNA, which aids the cytoplasmic release of plasmid DNA from the dendriplex, thus facilitating nuclear entry. PEGylated dendriplexes that led to higher transfection levels than non-PEGylated dendriplexes also showed higher cellular uptake. However, some G4- and G5-PEGylated dendriplexes showed low transfection levels, despite having higher cellular uptake. This demonstrates that the correlation between cellular uptake and transfection is complicated, cell line-dependent and governed mainly by internal cellular machinery, more than the extracellular factors. Similar increases in the levels of transfection after PEGylation of dendrimers have been reported before^16,23^. This is the first reported data showing the impact of PEG molecular weight on the transfection efficiencies of the different generations of DAB dendrimers on various cell lines.

## Methods

### Cell lines and reagents

Generations 3-, 4- and 5- diaminobutyric polypropylenimine (DAB) dendrimers were purchased from Symo-Chem (Eindhoven, The Netherlands). Methoxy PEG succinimidyl carboxymethyl esters of various molecular weights (2 kDa, 5 kDa and 10 kDa) came from Jenkem Technology (Plano, TX, USA). The expression plasmid encoding β-galactosidase (pCMVsport β-galactosidase) was obtained from Invitrogen (Paisley, UK). It was purified using an Endotoxin-free Giga Plasmid Kit (Qiagen, Hilden, Germany). Quanti-iT PicoGreen dsDNA reagent and tissue culture media were purchased from Life Technologies (Paisley, UK). Vectashield mounting medium containing 4′,6-diamidino-2-phenylindole (DAPI) was obtained from Vector Laboratories (Peterborough, UK). Label IT Fluorescein Nucleic Acid Labelling Kit was from Cambridge Biosciences (Cambridge, UK). Passive lysis buffer was obtained from Promega (Southampton, UK). All other materials were purchased from Sigma Aldrich (Poole, UK). Bioware PC-3M-luc-C6 human prostate adenocarcinoma and Bioware B16-F10-luc-G5 mouse melanoma were obtained from Caliper Life Sciences (Hopkinton, MA, USA), while A431 human epidermoid carcinoma, T98G human glioblastoma and DU145 human prostate carcinoma were purchased from the European Collection of Cell Cultures (Salisbury, UK).

### Synthesis of PEGylated diaminobutyric polypropylenimine (DAB) dendrimers

PEGylated dendrimers were synthesized following the one-step reaction of generation 3, 4- or 5- DAB dendrimers with the amine-reactive methoxy PEG succinimidyl carboxymethyl esters (molecular weights: 2 kDa, 5 kDa and 10 kDa) (M-PEG). Briefly, DAB dendrimers (10 mg) were dissolved in 5 mL of 50 mM sodium phosphate and 0.15 M sodium chloride buffer (pH 7.5). The amine-reactive M-PEG with various molecular weights were then added to each DAB dendrimer (10 mg) in 4 mole-excess (47.2 mg, 22.4 mg and 10.4 mg M-PEG 2 kDa reacting with respectively G3-, G4- and G5-dendrimers; 118 mg, 56 mg and 26 mg M-PEG 5 kDa reacting with respectively G3-, G4- and G5-dendrimers; 236 mg, 112 mg and 52 mg M-PEG 10 kDa reacting with respectively G3-, G4- and G5-dendrimers) for 8 hours at 25 °C with continuous stirring, yielding a total of 9 different PEGylated dendrimers. The final compounds were purified by dialysis against distilled water (1.8 L, changed twice) for 24 h at 25 °C, using dialysis tubings with molecular weight cut-offs of 3.5 kDa, 7 kDa and 12–14 kDa to remove excess M-PEG, before being freeze-dried. The PEGylation of the dendrimers was confirmed by ^1^H NMR spectroscopy, using a Jeol Oxford NMR AS 600 spectrometer (Peabody, MA, USA). The final products were designated as G3-PEG2K, G3-PEG5K, G3-PEG10K, G4-PEG2K, G4-PEG5K, G4-PEG10K, G5-PEG2K, G5-PEG5K and G5-PEG10K, depending on the generation of dendrimer and molecular weight of PEG.

### Cytotoxicity

The cytotoxicity of the PEGylated DAB dendrimers was assessed using a MTT assay. B16F10-Luc cells were seeded in quintuplicate at a density of 5 000 cells/well in 96-well plates for 24 hours before treatment. They were then incubated for 72 hours with various PEGylated dendrimers in concentrations ranging from 20 to 180 µg/mL, using untreated cells as negative controls and cells treated with 1% Triton X as positive controls. Absorbance was measured at 570 nm using a plate reader and the growth inhibitory concentration for 50% of the cells (IC_50_) was measured. Dose-response curves were fitted to percentage absorbance values to obtain IC_50_ values (n = 15).

### DNA condensation efficiency

The ability of PEGylated DAB dendrimers to successfully complex plasmid DNA was assessed using a PicoGreen assay, following the protocol provided by the supplier. PicoGreen reagent was diluted 200-fold in Tris-EDTA (TE) buffer (10 mM Tris, 1 mM EDTA, pH 7.5) just before the experiment. One mL of PEGylated DAB: DNA dendriplex at various dendrimer: DNA weight ratios (20:1, 10:1, 5:1, 2:1, 1:1 and 0.5:1) was added to one mL of the diluted PicoGreen reagent. The DNA concentration in the cuvette (10 µg/mL) remained constant throughout the whole experiment. The fluorescence intensity of PicoGreen in presence of the formed dendriplexes was measured at various times using a Varian Cary Eclipse fluorescence spectrophotometer (Palo Alto, CA, USA) (λ_exc_: 480 nm, λ_em_: 520 nm). Results were presented as percentages of DNA condensation.

### Size and zeta potential measurement of dendriplexes and dendrimers

The size and zeta potential of the PEGylated DAB dendriplexes were measured for various dendrimer: DNA weight ratios (20:1, 10:1, 5:1, 2:1, 1:1, and 0.5:1) respectively by photon correlation spectroscopy and laser Doppler electrophoresis, using a Malvern Zetasizer Nano-ZS at 37 °C (Malvern Instruments, Malvern, UK). The DNA concentration was 50 µg per 1 mL sample.

PEGylated and non-PEGylated dendrimers also had their size and zeta potential measured. For size measurement, dendrimer concentration was 5 mg per mL glucose 5% solution for non-PEGylated G5-dendrimers, and 10 mg per mL glucose 5% solution for all other PEGylated and non-PEGylated dendrimers. Size data were reported as size by volume. For zeta potential determination, the dendrimer concentration was 2 mg per 1 mL glucose 5% solution for all PEGylated and non-PEGylated dendrimers.

### Transfection

The transfection efficiency of the DNA complexed by PEGylated DAB dendrimers on various cancer cell lines was assessed using a plasmid DNA encoding β-galactosidase. To this end, B16F10-Luc, A431, T98G, PC3 and DU145 cells were seeded at a concentration of 2 000 cells/well in 96-well plates and incubated for 72 h at 37 °C in a 5% CO_2_ atmosphere. They were then treated with PEGylated dendriplexes in quintuplicate at various dendrimer: DNA weight ratios (20:1, 10:1, 5:1, 2:1, 1:1 and 0.5:1). Naked DNA was used as a negative control. Generations 3-, 4- and 5- DAB complexed to DNA served as positive controls, with a dendrimer: DNA weight ratio of 2:1 that was shown to be leading to the highest gene expression (Supplementary Fig. 5). DNA concentration (1 µg/ well) was kept constant throughout the experiment. The volume of the treatment per well was 0.2 mL. After treatment, the cells were incubated for 72 h at 37 °C before analysis. They were then lysed with 1X passive lysis buffer (PLB) (50 µL/well) for 20 min and then tested for β-galactosidase expression. Briefly, 50 µL of the assay buffer (2 mM magnesium chloride, 100 mM mercaptoethanol, 1.33 mg/mL O-nitrophenyl- β-D-galactosidase, 200 mM sodium phosphate buffer, pH 7.3) was added to each well containing the lysates, before being incubated for 2 h at 37 °C. The absorbance of the samples was subsequently read at 405 nm using a Multiskan Ascent plate reader (MTX Lab Systems, Bradenton, FL, USA).

### Cellular uptake

To determine whether PEGylation of dendrimers led to lower cellular uptake of the complexed DNA, ultimately resulting in lower transfection, B16F10-Luc cells were treated with two dendrimer: DNA weight ratios respectively resulting in high and low transfection.

Cellular uptake of the fluorescein-labelled DNA complexed to PEGylated dendrimers was qualitatively assessed using confocal microscopy. Labelling of plasmid DNA with the fluorescent probe fluorescein was performed using a Label IT Fluorescein Nucleic Acid Labelling kit, as described by the manufacturer. B16F10-Luc cells were seeded at a concentration of 100 000 cells per well on coverslips in 6-well plates and grown at 37 °C for 24 h. They were then treated with fluorescein-labelled DNA (2.5 µg/mL) at different dendrimer: DNA weight ratios of G3-PEG2K (20:1, 2:1), G3-PEG5K (20:1, 5:1), G3-PEG10K (20:1, 5:1), G4-PEG2K (10:1, 2:1), G4-PEG5K (20:1, 2:1), G4-PEG10K (20:1, 5:1), G5-PEG2K (5:1, 2:1), G5-PEG5K (5:1, 2:1) and G5-PEG10K (10:1, 2:1) for 1 h. G3-, G4- and G5- DAB dendrimers were used as positive controls and naked DNA or untreated cells were used as negative controls.

The cells were then washed three times with 3 mL phosphate buffered saline (PBS) before being fixed with 2 mL methanol for 10 min at 25 °C. Upon staining of the nuclei with Vectashield mounting medium containing DAPI, the cells were examined using a Leica TCS SP5 confocal microscope (Wetzlar, Germany). DAPI (staining the cell nuclei) was excited with the 405 nm laser line (emission bandwidth: 415–491 nm), while fluorescein (which labelled the DNA) was excited with the 514 nm laser line (emission bandwidth: 550–620 nm).

Quantification of cellular uptake was performed using flow cytometry. B16F10-Luc cells were seeded and treated at various dendrimer: DNA weight ratios of PEGylated dendrimers as described for confocal microscopy.

After 1 h incubation with the treatments, single cell suspensions were prepared (using 250 µL trypsin per well, followed by 500 µL medium per well once the cells have detached), before being analysed using a FACSCanto flow cytometer (BD, Franklin Lakes, NJ, USA). Ten thousand cells (gated events) were counted for each sample. Their mean fluorescence intensity was analysed with FACSDiva software (BD, Franklin Lakes, NJ, USA).

### Statistical analysis

Results were expressed as means ± standard error of the mean (S.E.M). Statistical significance was determined by analysis of variance (ANOVA) and 2-sample t-tests for pair-wise comparisons (Minitab software, State College, PE, USA). Differences were considered statistically significant for P values lower than 0.05.

### Availability of materials and data

All materials, data and protocols associated with this manuscript are promptly available to readers without undue qualifications in material transfer agreements.

## Conclusion

In this study, we have investigated *in vitro* a series of PEGylated DAB dendrimers, with various PEG molecular weight and dendrimer generations, for potential use as gene delivery systems. Our results indicated that G3-DAB dendrimer conjugated to low molecular weight PEG (2 kDa) drastically reduced the cytotoxicity of amine-terminated DAB dendrimers. G4-DAB conjugated to 2 and 5 kDa PEG also had its cytotoxicity reduced, but only at low concentrations (less than 20 µg/mL). These PEGylated dendrimers were still able to condense more than 70% of the DNA at the dendrimer: DNA weight ratios of 5:1 and higher (and even from 2:1 and above for the G4-PEGylated DAB dendrimers). At these dendrimer: DNA ratios, the size and zeta potential of the resulting dendriplexes were lower than that of unmodified dendriplexes. G3-DAB2K and G4-DAB2K dendriplexes at a dendrimer: DNA ratio of 20:1 increased transfection compared to the unmodified dendriplex on B16F10-Luc, A431, DU145, PC3-Luc cells lines, but not on T98G cell line. G4-DAB5K also increased transfection at a 20:1 ratio, but only on A431 cell line. PEGylated G3- and G4-DAB dendrimers may therefore serve as promising scaffolds to build more efficacious and versatile gene delivery systems for cancer therapy and should be further evaluated *in vivo*.

## Electronic supplementary material

Supplementary material
