# Supplementary material for: PEGylation of polypropylenimine dendrimers: effects on cytotoxicity, DNA condensation, gene delivery and expression in cancer cells
Source: Sci Rep. 2018 Jun 20;8:9410. doi: 10.1038/s41598-018-27400-6 (PMC6010408; doi:10.1038/s41598-018-27400-6)
Supplement: Supplementary file 1 — Supplementary material [file 41598_2018_27400_MOESM1_ESM.pdf]

PEGylation of polypropylenimine dendrimers: effects on cytotoxicity, DNA condensation, gene delivery and expression in cancer cells (Supplementary material)

Sukrut Somani[a], Partha Laskar[a], Najla Altwaijry[a], Paphitchaya Kewcharoenvong[a], Craig Irving[b], Gillian Robb[a], Benjamin S. Pickard[a], Christine Dufès[a*]

[a] *Strathclyde Institute of Pharmacy and Biomedical Sciences, University of Strathclyde, 161 Cathedral Street, Glasgow G4 0RE, United Kingdom*

[b] *Department of Pure and Applied Chemistry, University of Strathclyde, 295 Cathedral Street, Glasgow G1 1XL, United Kingdom*

* Corresponding author: Christine Dufès

Strathclyde Institute of Pharmacy and Biomedical Sciences, University of Strathclyde,

161 Cathedral Street, Glasgow G4 0RE, United Kingdom

Phone: 44 -141 548 3796

Fax: 44 -141 552 2562

E-mail: C.Dufes@strath.ac.uk

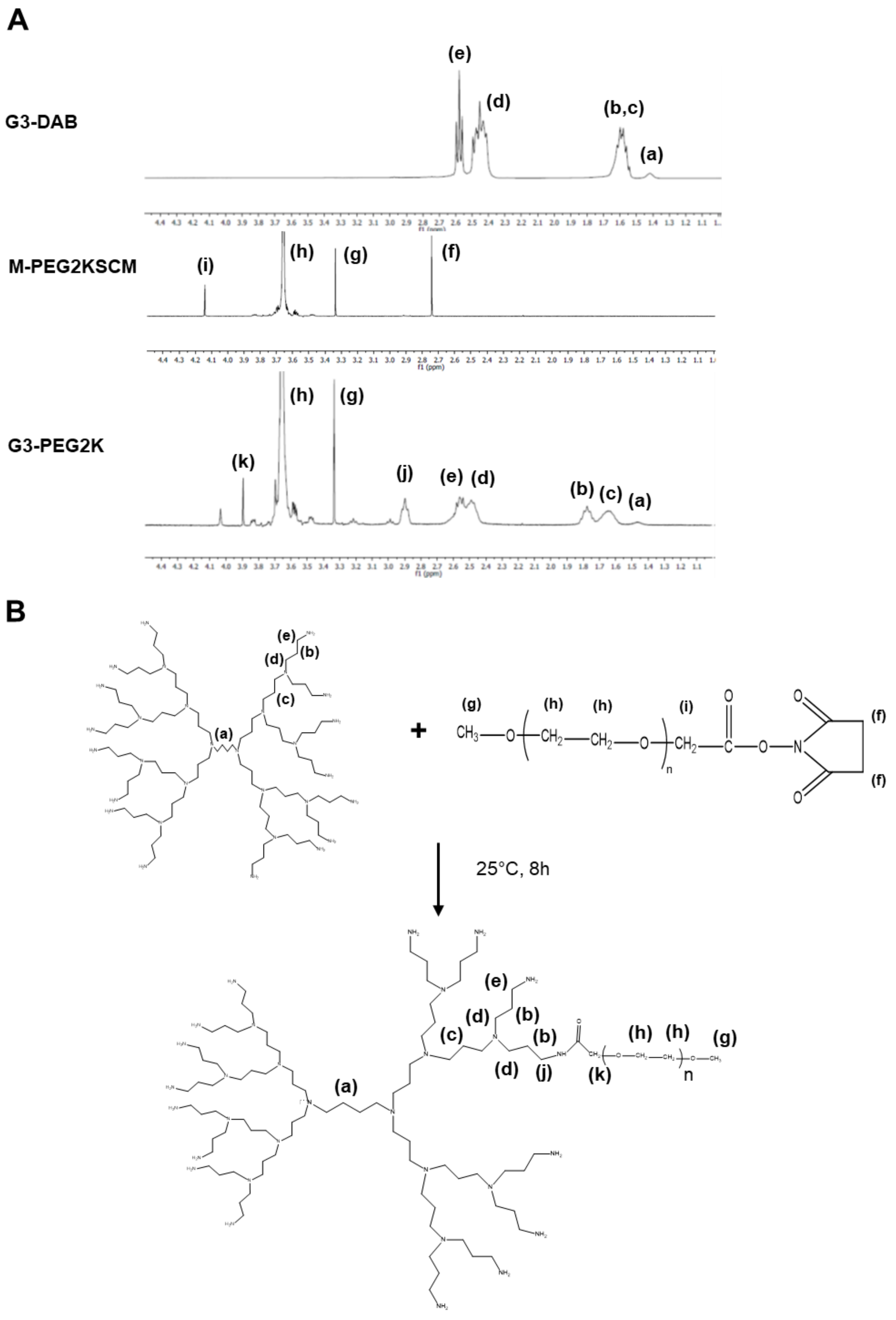

Supplementary Figure 1. A) ^{1}H NMR of G3 DAB, M-PEG2KSCM and G3-PEG2K in $D_2$0 at 600 MHz, B) Chemical reaction showing conjugation of M-PEG to G3-DAB via amide bond.

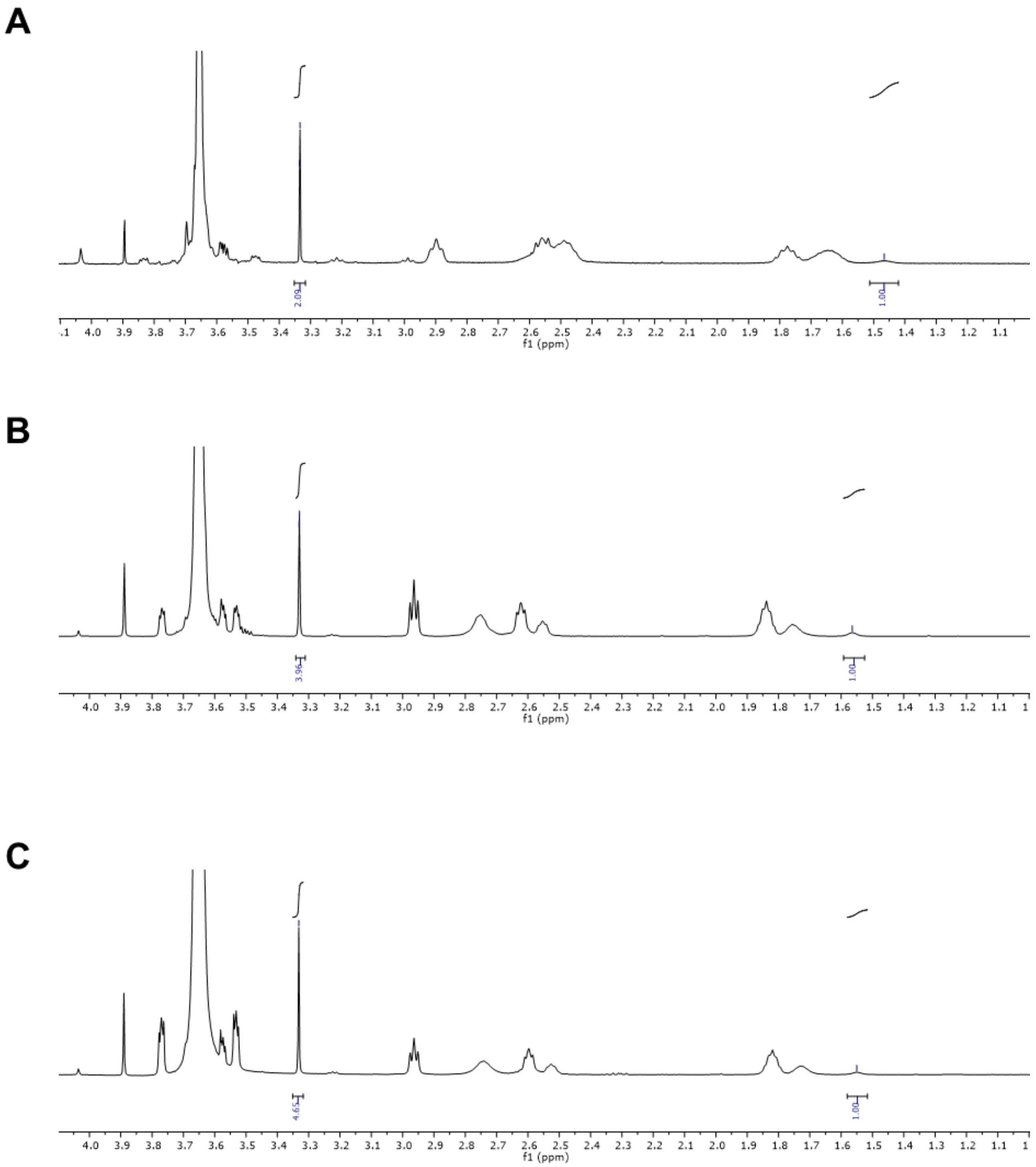

Supplementary Figure 2. ^{1}H NMR (600 MHz) of A) G3-PEG2K, B) G3-PEG5K and C) G3-PEG10K in $D_2$0.

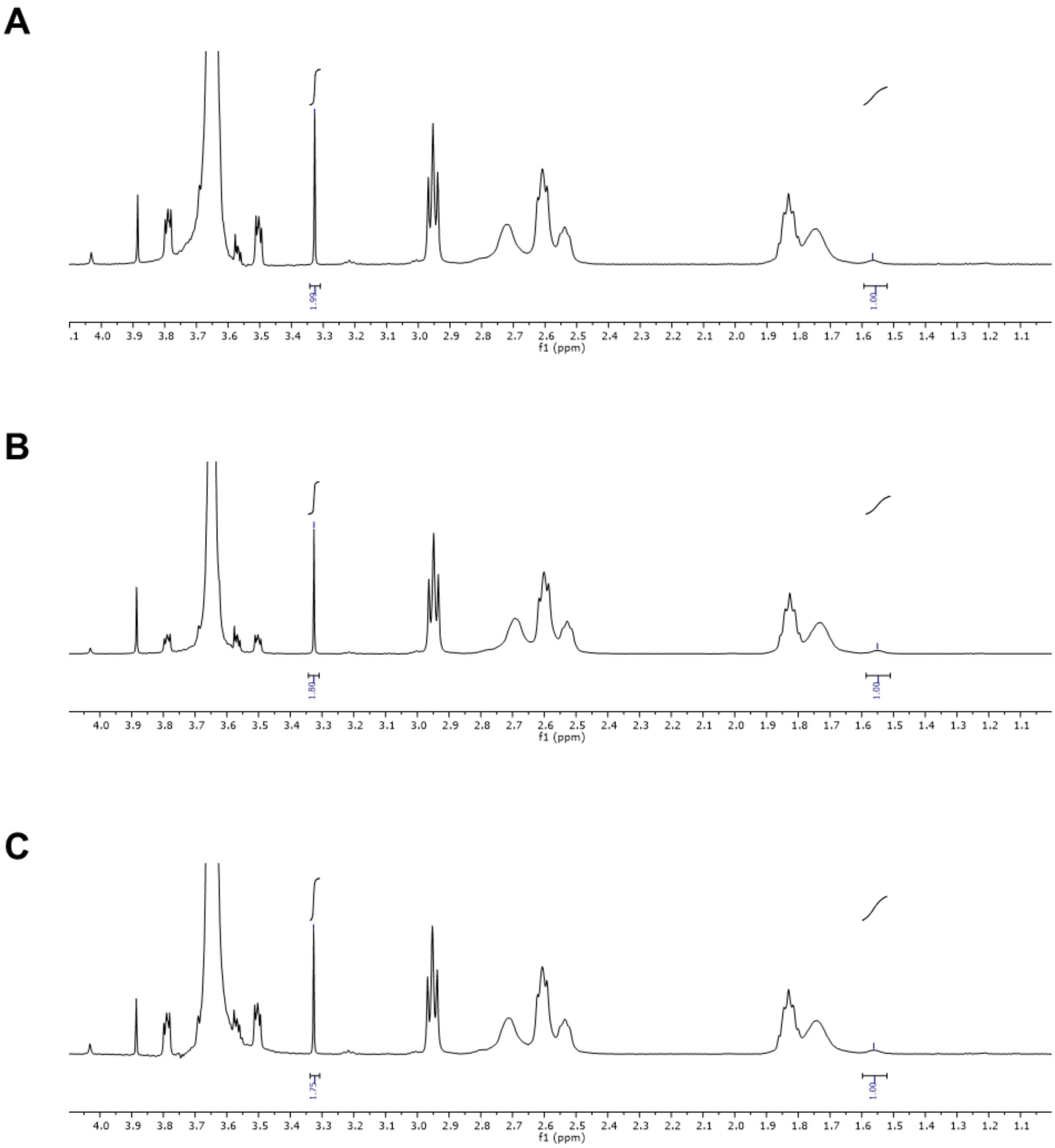

Supplementary Figure 3. ^{1}H NMR (600 MHz) of A) G4-PEG2K, B) G4-PEG5K and C) G4-PEG10K in D_20.

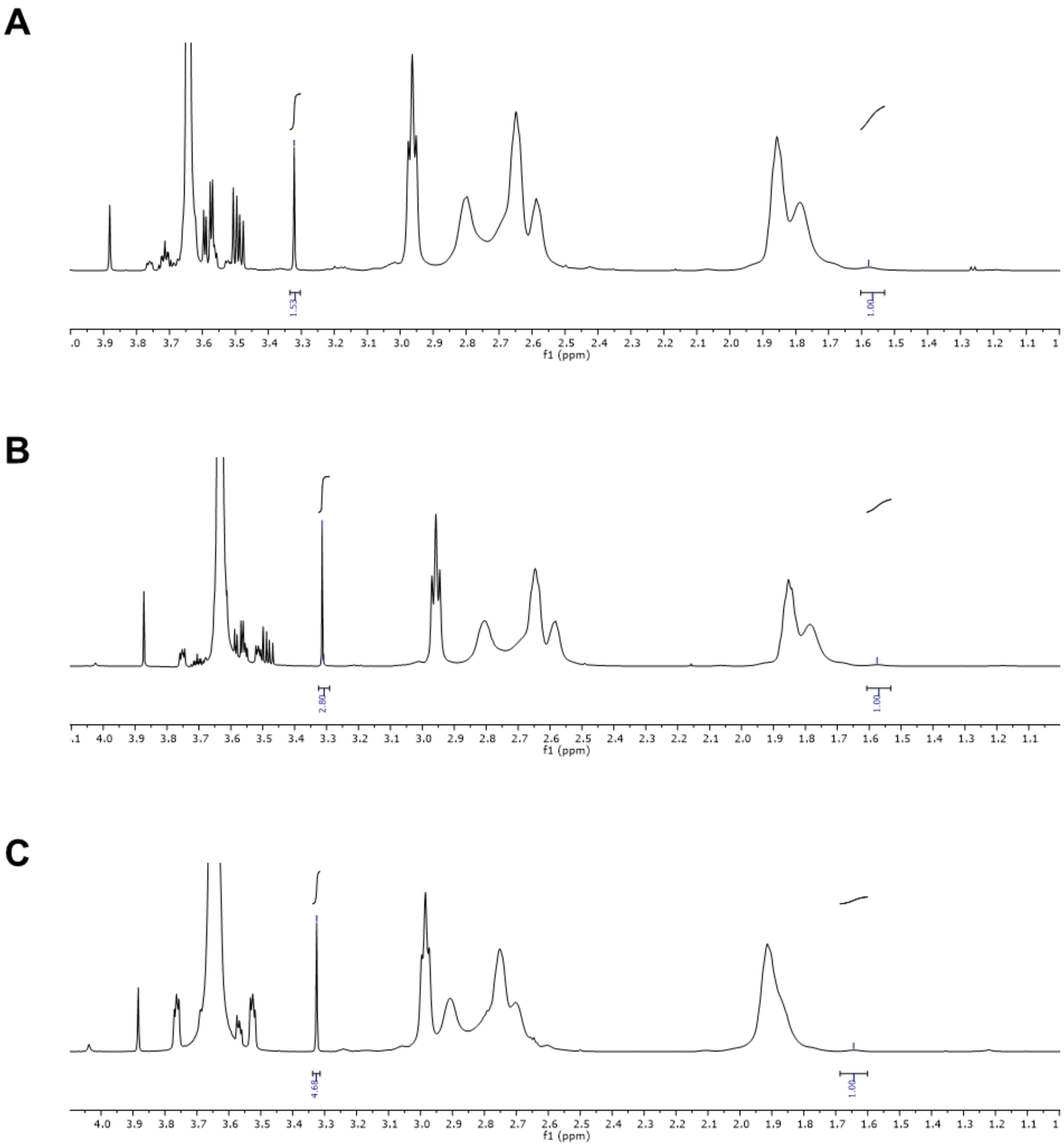

Supplementary Figure 4. ^{1}H NMR (600 MHz) of A) G5-PEG2K, B) G5-PEG5K and C) G5-PEG10K in $D_2$0.

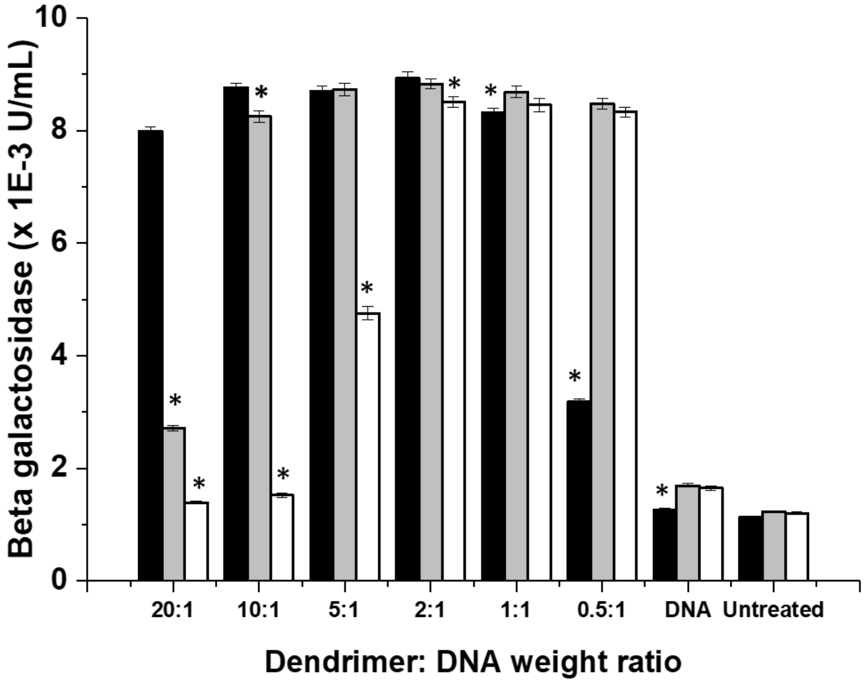

Supplementary Figure 5. Transfection efficacy of various generations of DAB dendriplexes at various dendrimer: DNA weight ratios in B16F10-Luc cancer cell line. Results are expressed as the mean ± SEM of three replicates (n=15) (Black: generation 3-DAB, grey: generation 4-DAB, white: generation 5-DAB). * : P <0.05 vs the highest transfection level for each dendrimer: DNA weight ratio.

Supplementary Table 1. Size of the various generations of DAB dendrimers and their PEGylated counterparts. Results are expressed as mean ± SEM (n= 4).

Dendrimer generation	Size (nm) (mean ± S.E.M.)			
	No PEG	PEG-2K	PEG-5K	PEG-10K
**G3**	1.73 ± 0.02	3.14 ± 0.16	4.49 ± 0.12	6.1 ± 0.13
**G4**	2.01 ± 0.04	2.23 ± 0.17	5.25 ± 0.72	6.54 ± 0.27
**G5**	2.53 ± 0.07	5.45 ± 0.38	4.45 ± 0.43	6.43 ± 0.24

Supplementary Table 2. Zeta potential of the various generations of DAB dendrimers and their PEGylated counterparts. Results are expressed as mean ± SEM (n= 4).

**Dendrimer generation**	**Zeta potential (mV) (mean ± S.E.M.)**			
	**No PEG**	**PEG-2K**	**PEG-5K**	**PEG-10K**
**G3**	13.05 ± 0.59	9.38 ± 0.77	13.08 ± 0.93	8.41 ± 0.42
**G4**	25.26 ± 0.78	16.06 ± 0.29	11.80 ± 0.32	8.74 ± 0.14
**G5**	41.50 ± 1.52	36.20 ± 0.20	21.38 ± 0.58	10.30 ± 0.22
